# Same-day emergency care: a retrospective observational study of the incidence and predictors of venous thromboembolism following hospital-based acute ambulatory medical care

**DOI:** 10.1016/j.jtha.2024.09.017

**Published:** 2025-01-01

**Authors:** Susan Shapiro, Jeannette Majert, Abubaker Obeidalla, Alex Clift, Sarah Havord, Angelin Jebamani, Charlotte Matejtschuk, Penney Clarke, Daniel Lasserson

**Affiliations:** 1 Department of Haematology, Oxford Haemophilia and Thrombosis Centre, Oxford University Hospitals NHS Foundation Trust, Oxford, United Kingdom; 2 Radcliffe Department of Medicine, University of Oxford, Oxford, United Kingdom; 3 Nuffield Department of Women’s and Reproductive Health, Medical Science Division, University of Oxford, Oxford, United Kingdom; 4 Department of Informatics, Oxford University Hospitals NHS Foundation Trust, Oxford, United Kingdom; 5 Department of Health Sciences, Warwick Medical School, University of Warwick, Coventry, United Kingdom; 6 Department of Geratology, Oxford University Hospitals NHS Foundation Trust, Oxford, United Kingdom

**Keywords:** ambulatory care, anticoagulant, emergency care, prevention, venous thromboembolism

## Abstract

**Background:**

Same-day emergency care (SDEC) is an expanding area of hospital acute medical care. It aims to minimize delays and manage medical emergency patients within the same day, enabling hospitalization to be avoided; the expectation is that the patients would have required inpatient hospitalization in the absence of the SDEC service. Venous thromboembolism (VTE) prevention is a key medical inpatient safety measure. Whether VTE prevention should be considered for SDEC patients is unknown.

**Objectives:**

To examine the incidence and predictors of VTE diagnosed within 90 days of SDEC assessment.

**Methods:**

Data were obtained from electronic health records of people who received SDEC at our hospital during a 5-year period (April 2016 to March 2021).

**Results:**

There were 40 045 attendance episodes by 33 715 individuals. Median age was 60 years (IQR, 41.0-76.0 years), and 55.2% were women. Three hundred forty-nine patients (0.9%) developed a VTE within 90 days of SDEC. Increased risk of VTE was associated with age more than 60 years, prior malignancy (adjusted odds ratio [OR], 4.12; 95% CI, 3.19-5.32; *P* < .0001), history of diseases of the circulatory system (adjusted OR, 2.92; 95% CI, 2.27-3.76; *P* < .0001), and having 1 or more additional SDEC attendances within 30 days (adjusted OR, 4.61; 95% CI, 3.65-5.82; *P* < .0001). In the 90 days prior to VTE diagnosis, 36.6% of patients had a separate inpatient admission in addition to SDEC. There was no association with completion of an electronic VTE risk assessment (adjusted OR, 0.96; 95% CI, 0.76-1.20).

**Conclusion:**

The incidence of VTE following SDEC is similar to that reported for symptomatic VTE in traditional medical inpatients without thromboprophylaxis.

## 1 Introduction

Same-day emergency care (SDEC) is the term used by the National Health Service (NHS) England to describe the provision of acute medical care for patients in a hospital ambulatory setting, enabling an inpatient medical admission to be avoided. It refers to the investigation, care, and treatment of patients who would have been admitted to hospital as inpatients in the absence of an SDEC service. The services are pre-dominantly run by specialists in acute medicine, and they achieve this by minimizing and removing delays in the patient pathway.

Ambulatory emergency care services aim to avoid admission and support early discharge. It is a rapidly expanding area of acute hospital care; a national audit in 2018 reported that 95% of UK hospitals had some form of ambulatory emergency care [1]. NHS digital data in 2018 showed that nonelective hospital “admissions” are now being disproportionately driven by “zero-day” admissions, ie, patients who are not actually admitted overnight to an acute bed [2]. Early evidence suggests that ambulatory services in the United Kingdom reduce hospital admissions with associated reduction in infections and deconditioning, improve patient experience, and have associated cost savings [3].

Hospital acute ambulatory services vary widely in design, size, and impact. However, the SDEC model, developed by the UK Emergency Care Network [4], has defined requirements and expectations. The NHS England Long Term Plan [2] mandated that the SDEC model of care should be embedded in all hospitals with a 24-hour emergency department, 12 hours a day, 7 days a week. SDEC patients may reattend when necessary on subsequent days for further investigation or review. While SDEC may also include patients who have had a brief overnight hospitalization and are discharged through SDEC the next day, as well as patients, followed up by SDEC after “early supported” discharge, this is rare, and the majority of care provision is for patients referred acutely to acute medicine. Although “hospital at home” models are being developed by some hospitals to further support patient care at home (currently termed “virtual wards” by NHS England), these are distinct from SDEC, where all processes of care are delivered on an acute hospital site [5]. Future research will be required to see if this unifying SDEC model supports patient care and experience [5]. A key part of that will be to find out whether core inpatient safety measures, such as venous thromboembolism (VTE) risk assessment and consideration of pharmacologic prophylaxis, are required for SDEC patients.

Hospitalization as a result of acute medical illness is associated with an increased risk of VTE, which is highest for the first 6 weeks after admission and persists for 3 months [6–9]. Thromboprophylaxis with low-molecular-weight heparin (LMWH) reduces the risk of VTE in acutely ill hospitalized medical patients by a relative risk (RR) of 0.49 (95% CI, 0.37-0.67), but it is associated with an increased risk of major bleeding (RR, 1.53; 95% CI, 0.8-2.92) [7,8,10–12]. National and international guidance advises that a VTE risk assessment should be undertaken on admission to the hospital for acutely unwell medical adult inpatients and for individual patients to be considered for pharmacologic thromboprophylaxis while an inpatient in order to reduce the risk of VTE [11,13]. The UK National Institute for Health and Care Excellence VTE prevention guidelines [11] advise that all acutely ill medical patients over the age of 16 years should be offered pharmacologic VTE prophylaxis for a minimum of 7 days if the risk of VTE outweighs the risk of bleeding. Hospitals have developed systems to support this, including electronic VTE risk assessments and electronic alerts for inpatients [14]. UK practice is currently that pharmacologic thromboprophylaxis is routinely given as an inpatient but is stopped on discharge, even if this is less than the minimum of 7 days stipulated by the UK National Institute for Health and Care Excellence guidelines [15,16]. Despite the fact that hospital ambulatory emergency care is increasing worldwide [17], no VTE prevention guidelines currently address this patient group, and the VTE risk of this patient group has not been reported.

Oxford University Hospitals NHS Foundation Trust is one of the largest teaching hospitals in the United Kingdom. It has one of the most established SDEC services, with 14 000 adults being reviewed in SDEC each year, which is around 25% of the total acute medical admissions. Our SDEC service accepts referrals from general practitioners, paramedics, and our emergency department [18]; it does not directly support inpatient postdischarge care as we have a separate service for this. Reflecting national VTE guidance, local VTE prevention guidance does not cover this group of patients currently, and it is left to the discretion of the treating physician. The aim of this study was to establish the incidence of VTE associated with SDEC attendance and key predictors of VTE risk. We retrospectively reviewed SDEC admissions over a 5-year period and ascertained those who had a new diagnosis of VTE in the 90 days following an SDEC attendance for a different primary diagnosis.

## 2 Methods

### 2.1 Study design and setting

This study used a retrospective observational design. Data were obtained from coding of electronic health records (EHRs) for adult patients who received SDEC at the Oxford University Hospitals NHS Foundation Trust (United Kingdom) over a 5-year period (April 1, 2016, to March 31, 2021); it was considered that the number of SDEC attendances over a 5-year period would be likely to provide a reasonable estimate of VTE risk and have sufficient numbers to look for predictors of VTE. The project was registered and approved as a clinical audit and service evaluation by Oxford Hospitals (approval number 6736) to analyze routinely collected data.

### 2.2 Selection of participants and data collection

EHRs of all patients who attended the adult Medical Acute Ambulatory Units at Oxford University Hospitals and received SDEC (ie, were not subsequently admitted as an inpatient at first assessment) were identified by the NHS Clinical Coding and Information Analytics Team. Based on clinical coding, data were extracted for primary diagnosis for SDEC attendance. In order to examine the association of VTE with SDEC, attendances for which the coding reason for SDEC attendance was new diagnosis of VTE were excluded. Based on clinical coding, data on the following comorbidities were extracted: obesity, malignancy, inflammatory polyarthropathy, noninfectious enteritis and colitis, cerebrovascular disease, ischemic heart disease, atrial fibrillation, personal history of diseases of the circulatory system (includes history of VTE but also previous stroke and rheumatic fever), and use of anticoagulation (Supplementary Table S1). Data from EHRs were extracted for patient demographics, blood results (hemoglobin, white cell count, platelet count, and C-reactive protein) at presentation to SDEC, whether an electronic VTE risk assessment had been completed, and death within 90 days of SDEC attendance.

Recurrent SDEC attendances during the 5-year period were recorded; recurrent presentations of a patient to SDEC within a 30-day period were counted as 1 “episode of care” and dated from the initial SDEC presentation within the 30-day period, as it was considered that this would reflect multiple attendances for the same medical issue. Data from coding were extracted for a new diagnosis of VTE between 48 hours and 90 days following SDEC attendance; VTEs diagnosed within 48 hours of SDEC attendance were considered to be a primary reason for SDEC attendance rather than the SDEC attendance being a risk factor for VTE and these patients were excluded. Missing data for the variables of age and sex were reviewed with source data verification. Data were extracted from EHR clinical codes, and it was not possible to account for undocumented assessments or diagnoses.

Electronic VTE risk assessments are available within EHRs, and completion is mandatory for hospitalized inpatients. The prescriber completes an assessment of thrombotic and bleeding risk factors, and then the electronic VTE risk assessment tool advises an outcome. For medical inpatients with thrombotic risk factors and no bleeding risk factors, the outcome recommendation is for prophylactic LMWH; if a patient does not have thrombotic risk factors or is already on anti-coagulation or has bleeding risk factors, then the outcome recommendation will NOT be for LMWH. The prescriber must agree or disagree with the recommendation; if the prescriber disagrees, they must complete a free text box with the reason. Information was extracted from EHRs on whether the VTE risk assessment was completed, prophylactic LMWH was recommended, and the prescriber agreed with this recommendation.

For patients who had a VTE, if the patient had more than 1 SDEC episode within 90 days of the VTE, then the VTE was linked to the first episode of care so that VTEs were not counted more than once. Data were extracted as to whether that patient had had inpatient hospitalizations within 90 days prior to the VTE diagnosis (either before or after the SDEC attendance).

### 2.3 Statistical analysis

All statistical analyses were performed in R (version 4.3.1, R Foundation for Statistical Computing) and Stata (version 17.0, StataCorp LLC). Descriptive statistics were used to describe the general study population and patients with a VTE diagnosis within 90 days of receiving SDEC. Data are presented as means or medians with SDs, IQRs, or 95% CIs, percentage of recruited patients, and odds ratios (ORs) with 95% CIs, unadjusted and adjusted.

Predictors associated with a VTE diagnosis were explored in a multivariable logistic regression model. The outcome variable of interest was the diagnosis of VTE within 90 days of SDEC. Published literature on risk factors for VTE in the general population and hospitalized inpatients was reviewed, and previously identified risk factors in these populations were adopted for the multivariable logistic regression model [19–21]. Demographic predictors included age and sex as well as comorbidities relating to obesity, malignancy, cerebrovascular disease, ischemic heart disease, atrial fibrillation, personal history of diseases of the circulatory system, inflammatory polyarthropathy, noninfectious enteritis, and colitis. Additionally, the use of anticoagulation, ≥1 additional SDEC episode within a 30-day period, and electronic VTE risk assessment completion were explored as predictors. Age categories were split as follows: under 40 years (reference category), 40 to 49 years, 50 to 59 years, 60 to 69 years, 70 to 79 years, and over 80 years. Additional SDEC attendances within a 30-day period were grouped as no additional attendances and ≥1 additional attendance. All other predictors were binary variables.

The choice of these predictors was guided by published literature; however, to reduce the risk of a type I error due to multiple comparison, a Bonferroni correction was applied, and a 2-tailed *P* value of ≤.004 was considered statistically significant. There were no missing data on the outcome or predictor variables, and hence, no imputation method was used. Sensitivity analyses were conducted to examine the multivariable model including all patient demographics and comorbidities, but with additional SDEC attendances within 30 days entered as a single continuous variable.

## 3 Results

In the 5-year period, the overall number of acute medical hospital-led ambulatory “episodes of care” was 45 332. Three thousand eight hundred eighty-one were excluded because patients were admitted directly from the ambulatory care medical assessment unit to inpatient wards and hence did not meet SDEC criteria. One thousand three hundred fifty-one records were excluded because the reason for SDEC attendance was new diagnosis of VTE; 57 were subsequently excluded because although the SDEC attendance was not initially coded as new VTE, the VTE was diagnosed within 48 hours and was therefore considered likely to be a primary reason for initial SDEC attendance. Overall, 40 045 “episodes of care” of 33 715 individual patients were included.

### 3.1 Characteristics of patients

SDEC was received by 33 715 individual patients over this 5-year period. For individuals who attended more than once during the 5-year period, the baseline characteristics at the first SDEC attendance were used, and these are shown in Table 1. Median age was 60 years (IQR, 41.0-76.0 years); 20 331 (55.2%) were women, and the major medical comorbidities are listed (Table 1).

### 3.2 Characteristics of SDEC episodes of care

The overall number of SDEC “episodes of care” during the 5-year period was 40 045. An SDEC episode of care is defined as ≥1 SDEC attendance in a 30-day period. The characteristics of the SDEC episode of care are based on the first attendance within that 30-day episode and are shown in Table 2. The top 5 reasons for SDEC attendance (primary diagnosis) based on coding groups were: Symptoms, Signs, and Abnormal Clinical and Lab Findings (32.9%); Respiratory system (13.4%); Circulatory system (11.8%); Genitourinary system (6.1%); and Musculoskeletal system (5.4%). Of note, the clinical code “Symptoms, Signs and Abnormal Clinical and Lab Findings” is used for an attendance when a definitive diagnosis has not been made (such as “shortness of breath” and “chest pain”); the patient may have further review and investigations on subsequent days. Blood results (hemoglobin, total white blood cell count, and platelet count data were available for 35 562 episodes and C-reactive protein data were available for 32 657 SDEC episodes) are shown in Table 2. Of those attending SDEC, 1598 (4.0%) died within 90 days. Cause of death was not extracted for this evaluation.

Electronic VTE risk assessments were completed for 41.3% of SDEC attendances. Of the completed VTE risk assessments, 25.2% had an outcome recommendation to prescribe prophylactic LMWH, and the prescriber accepted this recommendation; 30.0% had an outcome recommendation to prescribe prophylactic LMWH, and the prescriber disagreed with this recommendation; and for 44.8%, thromboembolic prophylaxis was not indicated (Table 2). It was not possible to electronically extract whether or not LMWH had actually been prescribed for all these patients. We randomly sampled 60 nonconsecutive individual EHRs in which the prescriber had agreed to prescribe prophylactic LMWH, and of those, 40 records showed that the prescriber had indeed prescribed prophylactic LMWH (for 1-3 days; Table 2).

### 3.3 Characteristics of individuals diagnosed with a VTE within 90 days following SDEC attendance

**Figure 1:**
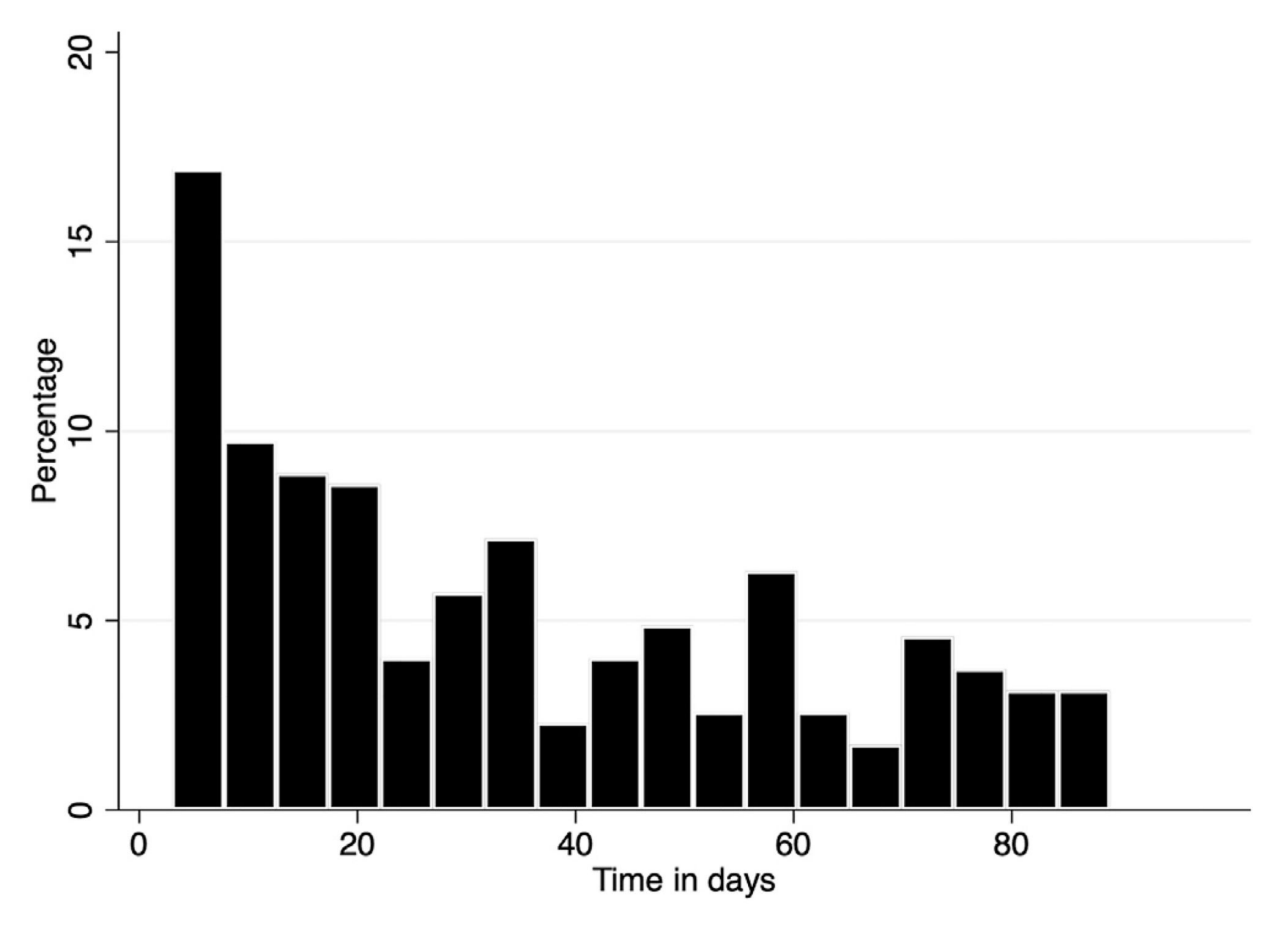
Histogram to show the time between same-day emergency care attendance and venous thromboembolism diagnosis.

Following a presentation to SDEC, 349 (0.9%) patients were subsequently diagnosed with VTE in the following 90 days. The median time between first SDEC episode and diagnosis of VTE was 29 days (IQR, 11-56.0 days; Table 3), and the overall time course is illustrated by a histogram (Figure 1). Of those diagnosed with a new VTE, 16.9% were diagnosed in the first 7 days, 35.2% between days 8 and 30, 28.9% between days 31 and 60, and 23.2% between days 61 and 90.

Mean age was 69.0 years, and 50.1% were men (Table 4). Cardiovascular comorbidities were common: circulatory disease, 28.7%; atrial fibrillation, 16.0%; heart disease, 14.9%; cerebrovascular disease, 2.0%; 16.3% were on anticoagulation, and 25.0% of patients had malignancy. The top 5 reasons for SDEC attendance (primary diagnosis) based on coding groups on the day of initial attendance were Symptoms, Signs, and Abnormal Clinical and Lab Findings (22.9%); Respiratory system (17.9%); Circulatory system (11.8%); Neoplasms including blood neoplasms (11.3%); and Musculoskeletal system (7.4%; Table 4). Less than half of the patients (38.4%) had an electronic VTE risk assessment completed in SDEC, and of those, the outcome recommendation was for LMWH prophylaxis in 71.7%, and prescribers agreed with the recommendation to prescribe LMWH prophylaxis in 27.7% (Table 4).

**Figure 2:**
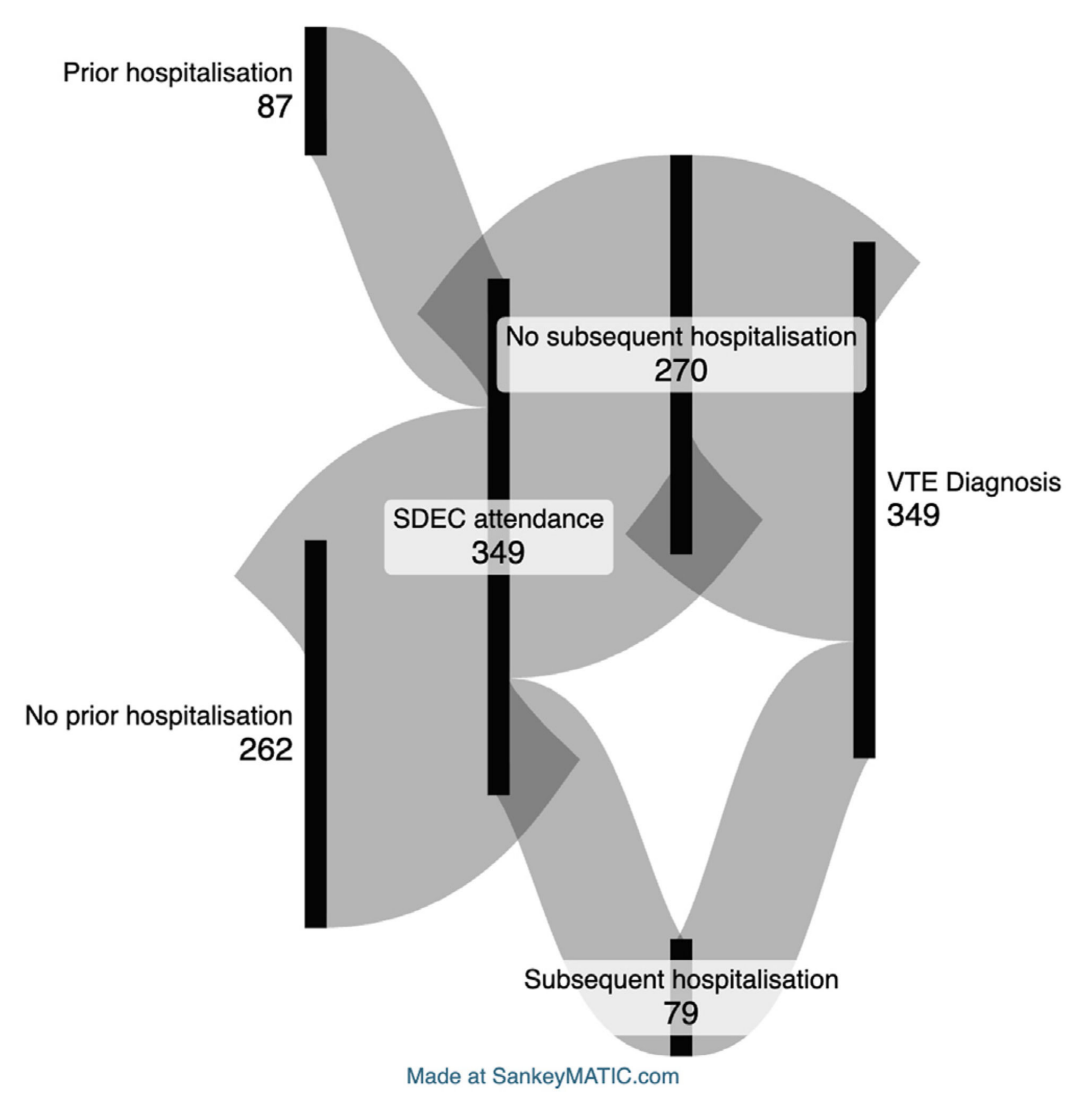
Sankey diagram to illustrate the flow of hospital attendances of patients diagnosed with venous thromboembolism (VTE) within 90 days of same-day emergency care (SDEC). Of the 349 patients diagnosed with VTE within 90 days of SDEC attendance, 87 had an additional prior inpatient hospitalization, and 79 had an additional subsequent inpatient hospitalization within the 90 days before VTE diagnosis.

Of the people diagnosed with a VTE within 90 days of SDEC, the majority had only 1 SDEC attendance prior to diagnosis; however, 32.7% had more than 1 SDEC attendance (Table 3, Supplementary Table S2); 36.6% also had at least 1 inpatient hospitalization within 90 days prior to VTE diagnosis (Table 3); 23.2% had an inpatient hospitalization before SDEC attendance, and 22.6% had a separate inpatient hospitalization after SDEC attendance but prior to VTE diagnosis (Supplementary Table S2). Figure 2 illustrates the patient pathway in a Sankey diagram. The majority of patients only had 1 inpatient hospitalization, but some patients had multiple inpatient hospitalizations (Supplementary Table S2). Of those diagnosed with a VTE, 73 (21.0%) died within 90 days of SDEC attendance, of whom 32 (43.8%) had malignancy.

### 3.4 Univariable and multivariable logistic regression to identify predictors for a VTE within 90 days of SDEC

The multivariable analysis demonstrated that patients over 60 years were most likely to develop a VTE following their SDEC episode (60-60 years: adjusted OR, 2.40; 95% CI, 1.52-3.78; *P* < .001; 70-79 years: adjusted OR, 3.04; 95% CI, 1.97-4.69; *P* < .0001; over 80 years: adjusted OR, 2.93; 95% CI, 1.89-4.54; *P* < .0001), whereas age less than 60 years was not associated with an increased likelihood (Table 5). Other factors, such as comorbidities relating to malignancy (adjusted OR, 4.12; 95% CI, 3.19-5.32; *P* < .0001) and history of diseases of the circulatory system (adjusted OR, 2.92; 95% CI, 2.27-3.76; *P* < .0001), were significantly associated with the development of a VTE. Also, 1 or more additional SDEC episodes within 30 days increased the likelihood of VTE diagnosis (adjusted OR, 4.61; 95% CI, 3.65-5.82; *P* < .0001). In comparison with this, prior anticoagulation or completion of an electronic VTE risk assessment were neither associated with an increased or decreased risk of developing a VTE. No association was demonstrated for variables such as sex, obesity, and comorbidities relating to atrial fibrillation, cerebrovascular diseases, ischemic heart disease, inflammatory polyarthropathy, noninfectious enteritis, and colitis. When entered into a separate multivariable model as a continuous single variable, additional SDEC attendances within a 30-day period was still a significant independent predictor of VTE diagnosis (adjusted OR, 1.80; 95% CI, 1.62-1.99; *P* < .0001; Supplementary Table S3).

## 4 Discussion

This retrospective study of 40 045 attendance episodes at an SDEC found that 0.9% were associated with a new VTE diagnosis in the following 90 days (excluding the first 48 hours). Significant risk factors for VTE included age more than 60 years, malignancy (adjusted OR, 4.12; 95% CI, 3.19-5.32; *P* < .0001), “personal history of prior diseases of the circulatory system” (adjusted OR, 2.92; 95% CI, 2.27-3.76; *P* < .0001), and more than 1 SDEC attendance within 30 days (adjusted OR, 4.61; 95% CI, 3.65-5.82; *P* < .0001).

Age and malignancy are well-recognized “strong” risk factors for VTE [22–24] but have not previously been described in the setting of SDEC. Although previous VTE is also a well-recognized risk factor for recurrent VTE, the coding of “personal history of prior diseases of the circulatory system,” which includes both history of previous VTE and previous stroke, is not sufficiently specific to attribute an association with prior VTE (or stroke) specifically in the setting of SDEC. Given that more than 1 SDEC attendance within 30 days was associated with increased risk of VTE, this could provide a potential VTE risk stratification for patients attending SDEC. Documented long-term (current) anticoagulation was not associated with altered risk of VTE in these patients (adjusted OR, 0.92; 95% CI, 0.65-1.30). Of note, it is not known whether anticoagulation was interrupted on admission, and at least some of these patients may have been at very high risk of VTE. Risk of VTE associated with prior use of anticoagulation has not been reported for general medical inpatients, as prior use of anti-coagulation excluded people from clinical trials of VTE prevention and clinical models of VTE inpatient risk [7,8,24]; however, in case series of hospitalized inpatients with COVID-19, prior use of anticoagulation appeared to reduce the risk of COVID-19–associated VTE [25,26].

Of the people who developed a new VTE following SDEC, 36.6% had also had an associated inpatient hospitalization within the 90 days prior to VTE diagnosis; 23.2% had an inpatient hospitalization before SDEC attendance, and 22.6% had a separate inpatient hospitalization after SDEC attendance but prior to VTE diagnosis. A recent United States observational study reported the rate of VTE as 71.8 per 1000 person-years during hospitalization compared with 1.4 per 1000 person-years for those people not recently hospitalized [9]. The rates during the first, second, and third months after discharge were 35.1, 11.3, and 5.2 per 1000 person-years, respectively [9]. The periods of hospitalizations will, therefore, have significantly increased the VTE risk of these SDEC patients, despite them likely receiving thrombo-prophylaxis during the period of actual hospitalization, but it is not possible to calculate this increased risk within this study.

The pattern of timing between SDEC and diagnosis of new VTE appears slightly different from that following inpatient hospitalization. The median time to VTE diagnosis from SDEC attendance was 29.0 days (IQR, 11.0-55.0 days), whereas the IMPROVE (International Medical Prevention Registry on Venous Thromboembolism) study of hospitalized medical patients had median time to event of 16 days [24], and the United States observational study reported the increased VTE risk significantly reducing with time following medical hospitalization: 36.5-fold increased (age-adjusted) hazard of VTE while in the hospital, and 17.4, 6.2, and 2.7 for the successive 1-month periods after discharge [9]. In comparison, patients appear to have the highest increased risk of VTE in the first few weeks following SDEC but an ongoing elevated risk after the first month, with minimal reduction in risk between months 2 and 3. The increased risk in the first few weeks following SDEC suggests that there is an increased VTE risk associated with the episode of medical illness that precipitates SDEC; the ongoing apparent persistent risk in months 2 and 3 could reflect higher baseline VTE risk in these patients due to comorbidities such as malignancy and/or could be a result of the periods of inpatient hospitalizations (in addition to SDEC) influencing the results.

Completion of an electronic VTE risk assessment is a requirement for hospitalized medical inpatients but not for acute medical hospital ambulatory care patients. Despite this, 41.3% of SDEC patients had an electronic VTE risk assessment completed, but there was no association between completion of an electronic VTE risk assessment and risk of VTE (adjusted OR, 0.96; 95% CI, 0.76-1.20). As electronic VTE risk assessments are not mandated in this patient group, it is not known why some patients had electronic VTE risk assessments completed; whether it was completed for patients whom clinicians considered to be at higher VTE risk or simply done as a routine safety-net in case the patient required inpatient hospitalization. Of those who had a VTE risk assessment recommendation for prophylactic LMWH, which was then “accepted” by the prescriber (10.4% of the cohort) on review of a subset of 60 records, a short course (1-3 days) of prophylactic LMWH was prescribed for two-thirds of these patients. Therefore, in contrast to our hospitalized inpatients, the vast majority had no thromboprophylaxis prescribed, and those that did usually only had a single dose.

Without an SDEC, these patients would be hospitalized as general medical inpatients for further management. In comparison with other published general medical cohorts, the rates of malignancy and obesity reported here are slightly lower (5.5% and 2.4% compared with 19.8% and 6.4%, respectively) [27]. The lower rate of active malignancy in the SDEC cohort may be a reflection that patients with known cancers have direct access to the local oncology day unit and may be reviewed/admitted directly; the lower rate of obesity could be due to missed body mass index recording and coding. However, this is a relatively unwell and comorbid cohort, with 4% mortality within 90 days of SDEC episode. For those who developed a VTE, 25% had active malignancy (type of cancer not known), and 21% died within 90 days of SDEC; 44% of those who died had active malignancy.

To the best of our knowledge, this is the first report of the risk of VTE associated with hospital-led acute medical ambulatory care. Without SDEC, these patients would have been admitted as medical inpatients and considered for thromboprophylaxis during their hospitalization. Locally, we have a robust reporting process for identifying hospital-associated VTEs that have occurred within 90 days of hospitalization [14], in order to comply with national requirements [28]. All VTEs occurring subsequent to hospitalization (excluding the first 24 hours) are reported. Over the same reporting period as our study, April 1, 2016, to March 31, 2021, there were 177 142 adult medical inpatient admissions (excluding medical/oncology day cases), of whom 983 were reported to have developed a hospital-associated thrombosis (0.55%). This is a similar incidence to the United States observational cohort study, which reported a cumulative incidence of VTE of 0.38% at 3 months following medical admission (no minimum duration of hospitalization) [9]. A higher 3-month incidence of symptomatic VTE following medical hospitalization has been reported in selected high VTE risk patients: 1% in the IMPROVE observational study (45% received pharmacologic thromboprophylaxis with heparin [unfractionated heparin or LMWH]) [24,29]; 1.55% in the PREVENT (the Prospective Evaluation of Dalteparin Efficacy for Prevention of Venous Thromboembolism in Immobilised Patients Trial) randomized control trial in the placebo group, reducing to 0.93% with prophylactic LMWH (RR, 0.70; 95% CI, 0.36-1.35) [7]; and 2.7% in the MEDENOX (Prophylaxis in Medical Patients with Enoxaparin trial) randomized control trial in the placebo group, reducing to 1.1% in the group who received 40 mg enoxaparin [8].

Albeit different methodology has been used, it is noteworthy that the risk of VTE in the 90 days following SDEC where people are not routinely VTE risk assessed or prescribed thromboprophylaxis appears to be approximately 50% higher than the risk of VTE in the 90 days following admission as an inpatient at our hospital when patients are routinely VTE risk assessed, and the majority prescribed thromboprophylaxis (0.9% VTE at 90 days vs 0.55%). The incidence is similar to that expected for medical inpatients not prescribed thromboprophylaxis. We have a strong local VTE prevention program for our medical inpatients, supported by national policies and a national VTE prevention program. All adult medical inpatients are assessed and considered for thromboprophylaxis. Hospital-led ambulatory care is a rapidly expanding area of medicine internationally; although patients reviewed in SDEC would have traditionally been hospitalized as inpatients, current policies and guidelines for VTE prevention do not cover these patients. The vast majority are not considered for thromboprophylaxis. This study highlights that SDEC patients are at relatively high risk of VTE, whether because of high baseline risk due to comorbidities, associated recent hospitalizations, the medical illness precipitating presentation to SDEC, and/or no routine consideration of thromboprophylaxis following attendance.

Thromboprophylaxis is associated with increased risk of bleeding, and the risk of bleeding depends on the bleeding risk of the individuals and the duration and type of prophylaxis. In the landmark trials for medical inpatients, LMWH was prescribed for 6 to 14 days, with increased risk of major bleeding (RR, 1.53; 95% CI, 0.8-2.92) [10,12], and the trials of extended postdischarge thromboprophylaxis for 28 to 45 days also demonstrated increased risk of International Society on Thrombosis and Haemostasis major or fatal bleeding (0.6% vs 0.3%; RR, 2.04; 95% CI, 1.42–2.91; *P* < .001) [30]. Any consideration of use of pharmacologic prophylaxis to reduce the risk of VTE in SDEC patients would need to be carefully balanced not only with practicality but also with increased risk of bleeding.

### 4.1 Strengths and limitations

The strengths of our single-center retrospective observational data are the large number of patient SDEC episodes, the depth of EHR extraction, and that there were no missing data on the outcome variable or variables that were included in the logistic regression. Limitations include that acute VTE diagnosis will have only been recorded for people representing and being diagnosed with VTE in Oxford; however, the majority of the population resides locally, so the likelihood of representing Oxford with complications of VTE is high [14]. The EHR is automatically updated with out-of-hospital deaths from a national NHS database, but cause of death is not known; it is, therefore, possible that undiagnosed VTE was the cause of death in some of these individuals and that these have not been accounted for.

Data on comorbidities were based on hospital clinical coding, so some diagnoses may have been missed, as coders include only those diagnoses considered directly relevant to the hospital admission. There is no single code for history of VTE, and so this could only be studied through the group code of “personal history of diseases of the circulatory system.” The reason for presentation to SDEC (primary diagnosis) was also extracted from clinical coding from the first presentation to SDEC; 32.9% did not have a specific diagnosis (code R: Symptoms, Signs, and Abnormal Clinical and Lab Findings) and a definitive diagnosis may have been made in some of these patients on subsequent SDEC attendances, but this data were not separately extracted. There were numerous codes, of which many were not specific to an actual diagnosis, so it was not feasible to categorize the reason for SDEC presentation into useful clinical categories to include in predictor analysis; however, 4 of the top 5 general categories for presentation were the same between the overall cohort and those who developed VTE with the exception of neoplasms, and indeed, known cancer on coding was found to be a predictive comorbidity on analysis. We excluded VTEs that were coded as the main reason for initial SDEC or that were diagnosed within the first 48 hours following SDEC, as it was considered that these were most likely to be the main reason for initial attendance but with delayed recognition or investigations, as opposed to VTE subsequent to an attendance for another diagnosis such as cellulitis or pneumonia; 48 hours was chosen instead of 24 hours, which is commonly chosen for inpatients as ambulatory pathway patients are often brought back the next day for scans. We were unable to include inpatient hospitalization as a variable in the logistic regression of predictors of VTE, as additional hospitalizations were extracted for those with a VTE (from 90 days before VTE diagnosis), and there was no equivalent time frame for hospitalizations for those without VTE. Of the 5-year period, the final year occurred during the COVID-19 pandemic, and it is known that COVID-19 hospitalization is associated with an increased risk of VTE [25]. However, although this may have increased the VTE rate associated with SDEC from March 2020 to April 2021, it is considered that its impact on the overall 5-year period data will be relatively small, partly because presenting with a primary Respiratory or Infection diagnosis code was not associated with increased risk of VTE and because of the published low rate of VTE associated with COVID-19 in nonhospitalized patients [31,32].

## 5 Conclusion

This study highlights for the first time that 0.9% of SDEC attendances are associated with a new VTE diagnosis in the following 90 days (excluding the first 48 hours). This is higher than the risk of VTE in our local general medical inpatients who receive inpatient thromboprophylaxis. The VTE risk is likely secondary not only to VTE risk associated with the medical condition precipitating SDEC but also to associated recent hospitalizations and potentially higher baseline risk of associated medical comorbidities. Although routine thromboprophylaxis would be unlikely to be justified unless higher risk groups can be robustly identified, given the expansion of hospital-led acute ambulatory medical care worldwide, this is an important area for future research.

## Supplementary Material

The online version contains supplementary material available at https://doi.org/10.1016/j.jtha.2024.09.017

Supplementary material

## Figures and Tables

**Table 1:** Baseline characteristics of patients attending same-day emergency care.

Characteristics	Values
No. of patients, *N*	33 715
Age (y), median (IQR)	60 (41.0-76.0)
Sex, *n* (%)	
Men	15 096 (44.8)
Women	18 619 (55.2)
Weight (kg), median (IQR)	72.6 (65.0-85.0)
Comorbidities, *n* (%)	
Malignant neoplasms	1847 (5.5)
Obesity	801 (2.4)
Personal history of diseases of the circulatory system	3164 (9.4)
Ischemic heart disease	3458 (10.3)
Cerebrovascular disease	635 (1.9)
Inflammatory polyarthropathy	1843 (5.5)
Noninfections enteritis and colitis	547 (1.6)
Atrial fibrillation and flutter	4257 (12.6)
History of long-term (current) use of anticoagulation, *n* (%)	3183 (9.4)

For patients who attended more than once during the 5-year period, the baseline characteristics at the first same-day emergency care attendance are shown. Information for weight was available for 13 810 patients (41.0%).

**Table 2:** Characteristics of same-day emergency care episodes.

Characteristics	Values
Overall no. of SDEC care episodes, *n* (%)	40 045 (100)
Duration of SDEC attendance (h), median (IQR)/mean (SD)	5.0 (4.0-8.0)/11.6 (34.9)
No. of additional SDEC attendances within each 30-d SDEC care episode, n (%)	
1 or more additional attendances	3635 (9.1)
1 additional attendance	2950 (81.2)
2 additional attendances	453 (12.5)
3 additional attendances	125 (3.4)
4 additional attendances	50 (1.4)
5 additional attendances	32 (0.9)
6 additional attendances	14 (0.4)
7 additional attendances	5 (0.1)
8 additional attendances	6 (0.1)
Reason (primary diagnosis) for SDEC attendance, ICD code group, n (%)	
Infectious and parasitic diseases (A)	903 (2.3)
Infectious and parasitic diseases (B)	760 (1.9)
Neoplasms (C)	384 (1.0)
Neoplasms, blood, blood-forming organs (D)	1418 (3.5)
Endocrine, nutritional, and metabolic diseases (E)	1907 (4.8)
Mental and behavioral disorders (F)	390 (1.0)
Nervous system (G)	955 (2.4)
Eye and adnexa (H)	405 (1.0)
Circulatory system (I)	4733 (11.8)
Respiratory system (J)	5351 (13.4)
Digestive system (K)	1718 (4.3)
Skin and subcutaneous tissue (L)	1913 (4.8)
Musculoskeletal system (M)	2154 (5.4)
Genitourinary system (N)	2437 (6.1)
Pregnancy, childbirth, and puerperium (O)	46 (0.1)
Conditions originating in the perinatal period (Q)	11 (0.03)
Symptoms, signs, and abnormal clinical and laboratory findings (R)	13 193 (32.9)
Injury, poisoning, and other consequences of external causes (S)	500 (1.3)
Injury, poisoning, and other consequences of external causes (T)	335 (0.8)
Emergency codes (U)	381 (0.9)
Factors influencing health status (Z)	151 (0.4)
Blood results, median (IQR)	
Hemoglobin (g/L)	133.0 (120.0-145.0)
White blood cell count (×10^9^/L)	7.8 (6.2-10.0)
Platelet count (×10^9^/L)	254 (208.0-310.0)
C-reactive protein (mg/L)	6.5 (1.8-30.6)
Electronic VTE RA	
VTE RA completed	16 525 (41.3)
Recommendation to prescribe LMWH; outcome agreed	4157 (10.4)
Recommendation to prescribe LMWH; outcome not agreed	4960 (12.4)
Recommendation: thromboembolic prophylaxis not indicated	7408 (18.5)
Review of a random subset of patients for whom VTE RA advised LMWH and prescriber had agreed with the outcome, *n*	60
Prescribed LMWH (as agreed in VTE RA outcome)	40
1 d of LMWH injections	27
2 d of LMWH injections	11
3 d of LMWH injections	2
Not prescribed LMWH (contrary to VTE RA outcome)	20
Death within 90 d of SDEC, *n* (%)	1598 (4.0)

An SDEC episode of care is defined as 1 or more SDEC attendance in a 30-day period. The characteristics are based on the first attendance within that 30-day episode. The characteristics, except for the blood results, are shown for all 43 906 episodes. Hemoglobin, white cell count, and platelet count were known for 35 562 (88.8%) attendances, and C-reactive protein for 32 657 (81.6%) attendances. ICD, International Classification of Diseases; LMWH, low-molecular-weight heparin; RA, risk assessment; SDEC, same-day emergency care; VTE, venous thromboembolism.

**Table 3:** Key features of venous thromboembolism diagnosed within 90 days following same-day emergency care attendance.

Key features	Values
Overall no. of SDEC episodes, *n* (%)	40 045 (100)
VTE within 90 d, *n* (%)	349 (0.9)
Time between diagnosis of VTE and first SDEC episode (d), median (IQR)/ mean (SD)	29.0 (11.0-56.0)/34.2 (25.5)
Diagnosed in first 7 d, *n* (%)	59 (16.9)
Diagnosed between d 8 and 30, *n* (%)	123 (35.2)
Diagnosed between d 31 and 60, *n* (%)	101 (28.9)
Diagnosed between d 61 and 90, *n* (%)	66 (18.9)
No. of patients who had 1 or more additional SDEC attendances within 30-d episode of care, n (%)	114 (32.7)
No. of patients who had at least 1 hospitalization within 90 d of VTE (either before or after SDEC attendance), n (%)	128 (36.6)
Death within 90 d of SDEC and VTE, *n* (%)	73 (21.0)

SDEC, same-day emergency care; VTE, venous thromboembolism.

**Table 4:** Characteristics of patients with a new venous thromboembolism diagnosis within 90 days following same-day emergency care.

Characteristics	Values
VTE within 90 d, *n* (%)	349 (100)
Age (y), median (IQR)/mean (SD)	72 (60.0-81.0)/69.0 (16.5)
Under 40 y, *n* (%)	29 (8.3)
40-49 y, *n* (%)	19 (5.4)
50-59 y, *n* (%)	36 (10.3)
60-69 y, *n* (%)	60 (17.2)
70-79 y, *n* (%)	99 (28.4)
Over 80 y, *n* (%)	106 (30.4)
Sex, n (%)	
Men	175 (50.1)
Women	174 (49.9)
Comorbidities, *n* (%)	
Malignant neoplasms	86 (25.0)
Obesity	12 (3.4)
Personal history of diseases of the circulatory system	100 (28.7)
Ischemic heart disease	52 (14.9)
Cerebrovascular disease	7 (2.0)
Inflammatory polyarthropathy	19 (5.4)
Noninfections enteritis and colitis	6 (1.7)
Atrial fibrillation and flutter	56 (16.0)
History of long-term (current) use of anticoagulation	57 (16.3)
Reason for SDEC, *n* (%)	
Infectious and parasitic diseases (A)	5 (1.4)
Infectious and parasitic diseases (B)	3 (0.9)
Neoplasms (C)	18 (5.2)
Neoplasms, blood, blood-forming organs (D)	21 (6.1)
Endocrine, nutritional, and metabolic diseases (E)	17 (4.9)
Mental and behavioral disorders (F)	4 (1.1)
Nervous system (G)	8 (2.3)
Eye and adnexa (H)	2 (0.6)
Circulatory system (I)	41 (11.8)
Respiratory system (J)	62 (17.9)
Digestive system (K)	16 (4.6)
Skin and subcutaneous tissue (L)	15 (4.3)
Musculoskeletal system (M)	26 (7.4)
Genitourinary system (N)	20 (5.7)
Pregnancy, childbirth, and puerperium (O)	1 (0.3)
Conditions originating in the perinatal period (Q)	0
Symptoms, Signs and Abnormal Clinical and Lab Findings (R)	80 (22.9)
Injury, poisoning, and other consequences of external causes (S)	1 (0.3)
Injury, poisoning, and other consequences of external causes (T)	5 (1.4)
Emergency codes (U)	2 (0.6)
Factors influencing health status (Z)	1 (0.3)
VTE RA, *n* (%)	349 (100)
VTE RA completed	134 (38.4)
Recommendation to prescribe LMWH; outcome agreed	37 (10.6)
Recommendation to prescribe LMWH; outcome not agreed	59 (16.9)
Recommendation: thromboembolic prophylaxis not indicated	38 (10.9)

LMWH, low-molecular-weight heparin; RA, risk assessment; SDEC, same-day emergency care; VTE, venous thromboembolism.

**Table 5:** Univariable and multivariable logistic regression to identify predictors for a venous thromboembolism within 90 days of same-day emergency care.

Covariate	Univariable analysis, OR (95% CI)	*P* value	Multivariable analysis,^a^ OR (95% CI)	*P* value
Age				
Under 40 y	1.00 (reference)		1.00 (reference)	
40-49 y	1.25 (0.70-2.24)	.444	1.13 (0.63-2.02)	.678
50-59 y	1.86 (1.14-3.04)	.013	1.50 (0.91-2.48)	.110
60-69 y	3.07 (1.97-4.79)	<.0001	2.40 (1.52-3.78)	<.0001
70-79 y	4.17 (2.75-6.32)	<.0001	3.04 (1.97-4.69)	<.0001
Over 80 y	3.78 (2.51-5.71)	<.0001	2.93 (1.89-4.54)	<.0001
Sex				
Men	1.00 (reference)		1.00 (reference)	
Women	0.78 (0.64-0.97)	.028	0.89 (0.71-1.10)	.304
History of long-term (current) use of anticoagulation	1.50 (1.13-2.00)	.005	0.92 (0.65-1.30)	.651
Obesity	1.28 (0.71-2.28)	.403	1.54 (0.85-2.77)	.146
Malignant neoplasms	5.29 (4.13-6.78)	<.0001	4.12 (3.19-5.32)	<.0001
Atrial fibrillation or flutter	1.13 (0.85-1.51)	.386	0.73 (0.52-1.03)	.076
Cerebrovascular disease	0.95 (0.45-2.02)	.907	0.62 (0.29-1.34)	.228
History of disease of the circulatory system	3.32 (2.62-4.19)	<.0001	2.92 (2.27-3.76)	<.0001
Ischemic heart disease	1.29 (0.96-1.74)	.085	0.91 (0.66-1.24)	.555
Inflammatory polyarthropathy	0.89 (0.56-1.42)	.644	0.72 (0.45-1.15)	.178
Noninfectious enteritis and colitis	1.00 (0.44-2.25)	.993	0.97 (0.42-2.19)	.943
VTE risk assessment completed	0.88 (0.71-1.10)	.274	0.96 (0.76-1.20)	.734
1 or more additional SDEC attendances within 30 d				
No additional attendances	1.00 (reference)		1.00 (reference)	
1 or more additional attendances	4.98 (3.97-6.25)	<.0001	4.61 (3.65-5.82)	<.0001

OR, odds ratio; SDEC, same-day emergency care; VTE, venous thromboembolism.

a Adjusted for baseline characteristics of age, sex, and comorbidities.
