# Supplementary material for: Same-day emergency care: a retrospective observational study of the incidence and predictors of venous thromboembolism following hospital-based acute ambulatory medical care
Source: J Thromb Haemost. Author manuscript; Available in PMC 2026 Jan 20. (PMC7618650; doi:10.1016/j.jtha.2024.09.017)
Supplement: Supplementary material [file EMS211904-supplement-Supplementary_material.docx]

**Supplementary Table S1. ICD-10 codes used to identify VTE and comorbidities.**

**VTE diagnosis codes**

I26.0 Pulmonary embolism with mention of acute cor pulmonale

I26.9 Pulmonary embolism without mention of acute cor pulmonale

I80.1 Phlebitis and thrombophlebitis of femoral vein

I80.2 Phlebitis and thrombophlebitis of other deep vessels of the lower extremities

I82.2 Embolism and thrombosis of vena cava

**Codes for identifying comorbidities**

C00-C97 Malignant neoplasms

E66 Obesity

I20-25 Ischaemic heart disease (angina, acute myocardial infarction, subsequent myocardial infarction, chronic ischaemic heart disease)

I48 Atrial fibrillation and flutter

I60-I69 Cerebrovascular disease (cerebral infarction, intracerebral haemorrhage, subarachnoid haemorrhage)

K50-K52 Non-infectious enteritis and colitis

M05-M14 Inflammatory polyarthopathy

Z86.7 Personal history of diseases of the circulatory system (includes conditions classifiable I00-I99; that is pulmonary embolism, deep vein thrombosis, cerebrovascular disease, previous hypertension, rheumatic fever)

Z92.1 Personal history of long-term (current) use of anticoagulants

**Supplementary Table S2. For the 349 patients who were diagnosed with a VTE within 90 days of SDEC attendance; this table shows details of the number of additional SDEC attendances within the 30 day episode of care, and the additional associated inpatient hospitalisation episodes (timing, number, duration) within 90 days of VTE diagnosis.** Abbreviations: interquartile range, IQR; low molecular weight heparin, LMWH; number, n; risk assessment, RA; same day emergency care, SDEC; standard deviation, SD; venous thromboembolism, VTE.

| Number of patients who had 1 or more additional SDEC attendances within 30 day episode of care, n (%) | 114 (32.7) |
| --- | --- |
| One additional attendance | 89 (25.5) |
| Two additional attendances | 16 (4.6) |
| Three additional attendances | 6 (1.7) |
| Four additional attendances | 1 (0.3) |
| Five additional attendances | 1 (0.3) |
| Six additional attendances | 0 |
| Seven additional attendances | 0 |
| Eight additional attendances | 1 (0.3) |
|  |  |
| Number of patients who had hospitalisation (inpatient admission) prior to SDEC (within 90 days of VTE), n (%) | 87 (23.2) |
| One admission | 67 (19.2) |
| Two admissions | 15 (4.3) |
| Three admissions | 3 (0.8) |
| Four admissions | 2 (0.6) |
| Duration of hospitalisation prior to SDEC in days, median (IQR) / mean (SD) |  |
| First hospitalisation | 2.0 (1.0; 6.0) / 4.2 (6.4) |
| Second hospitalisation | 2.0 (1.0; 6.0) / 3.3 (3.3) |
| Third hospitalisation | 3.0 (2.0; 4.0) / 3.2 (1.9) |
| Fourth hospitalisation | 9.5 (8.0; 11.0) / 9.5 (2.1) |
| Number of patients who had hospitalisation (inpatient admission) between SDEC episode and VTE, n (%) | 79 (22.6) |
| One admission | 55 (15.7) |
| Two admissions | 19 (5.4) |
| Three admissions | 3 (0.8) |
| Four admissions | 2 (0.6) |
| Duration of hospitalisation between SDEC episode and VTE in days, median (IQR) / mean (SD) |  |
| First hospitalisation | 3.0 (1.0; 7.0) / 5.1 (5.8) |
| Second hospitalisation | 3.0 (1.0; 6.0) / 4.2 (3.7) |
| Third hospitalisation | 5.0 (2.0; 8.0) / 6.8 (6.2) |
| Fourth hospitalisation | 1.0 (1.0; 1.0) / 1 (0.0) |

**Supplementary Table S3.** **Sensitivity analysis with variable of additional same day emergency care (SDEC) attendances within a 30-day period treated as a single continuous variable instead of a categorical variable.** Abbreviations: confidence interval, CI; odds ratio, OR; venous thromboembolism, VTE. *Adjusted for baseline characteristics: Age, gender, and co-morbidities.

| **Covariate** | **Univariable analysis, OR and 95% CI** | **P-value** | **Multivariable analysis*, OR and 95% CI** | **P-value** |
| --- | --- | --- | --- | --- |
| One or more additional SDEC attendances within 30 days |  |  |  |  |
| - One or more additional attendances | 1.76 (1.60 to 1.94) | <0.0001 | 1.80 (1.62 to 1.99) | <0.0001 |

STROBE Statement—Checklist of items that should be included in reports of ***cross-sectional studies***

|  | **Item No** | **Recommendation** | **Page No** |
| --- | --- | --- | --- |
| **Title and abstract** | 1 | (*a*) Indicate the study’s design with a commonly used term in the title or the abstract | 1 |
|  |  | (*b*) Provide in the abstract an informative and balanced summary of what was done and what was found | 2 |
| **Introduction** | | | |
| Background/rationale | 2 | Explain the scientific background and rationale for the investigation being reported | 3-4 |
| Objectives | 3 | State specific objectives, including any prespecified hypotheses | 2, 4 |
| **Methods** | | | |
| Study design | 4 | Present key elements of study design early in the paper | 4 |
| Setting | 5 | Describe the setting, locations, and relevant dates, including periods of recruitment, exposure, follow-up, and data collection | 4 |
| Participants | 6 | (*a*) Give the eligibility criteria, and the sources and methods of selection of participants | 4,5 |
| Variables | 7 | Clearly define all outcomes, exposures, predictors, potential confounders, and effect modifiers. Give diagnostic criteria, if applicable | 5 |
| Data sources/ measurement | 8* | For each variable of interest, give sources of data and details of methods of assessment (measurement). Describe comparability of assessment methods if there is more than one group | 5 |
| Bias | 9 | Describe any efforts to address potential sources of bias | 6 |
| Study size | 10 | Explain how the study size was arrived at | 4 |
| Quantitative variables | 11 | Explain how quantitative variables were handled in the analyses. If applicable, describe which groupings were chosen and why | 6 |
| Statistical methods | 12 | (*a*) Describe all statistical methods, including those used to control for confounding | 6 |
|  |  | (*b*) Describe any methods used to examine subgroups and interactions | 6 |
|  |  | (*c*) Explain how missing data were addressed | 6 |
|  |  | (*d*) If applicable, describe analytical methods taking account of sampling strategy | NA |
|  |  | (*e*) Describe any sensitivity analyses | 6 |
| **Results** | | | |
| Participants | 13* | (a) Report numbers of individuals at each stage of study—eg numbers potentially eligible, examined for eligibility, confirmed eligible, included in the study, completing follow-up, and analysed | 7 |
|  |  | (b) Give reasons for non-participation at each stage | 7 |
|  |  | (c) Consider use of a flow diagram |  |
| Descriptive data | 14* | (a) Give characteristics of study participants (eg demographic, clinical, social) and information on exposures and potential confounders | 7, 8 |
|  |  | (b) Indicate number of participants with missing data for each variable of interest | 6 |
| Outcome data | 15* | Report numbers of outcome events or summary measures | 7, 8 |
| Main results | 16 | (*a*) Give unadjusted estimates and, if applicable, confounder-adjusted estimates and their precision (eg, 95% confidence interval). Make clear which confounders were adjusted for and why they were included | 8, 9 |
|  |  | (*b*) Report category boundaries when continuous variables were categorized | 6 |
|  |  | (*c*) If relevant, consider translating estimates of relative risk into absolute risk for a meaningful time period | NA |
| Other analyses | 17 | Report other analyses done—eg analyses of subgroups and interactions, and sensitivity analyses | 8, 9 |
| **Discussion** | | | |
| Key results | 18 | Summarise key results with reference to study objectives | 9 |
| Limitations | 19 | Discuss limitations of the study, taking into account sources of potential bias or imprecision. Discuss both direction and magnitude of any potential bias | 12, 13 |
| Interpretation | 20 | Give a cautious overall interpretation of results considering objectives, limitations, multiplicity of analyses, results from similar studies, and other relevant evidence | 9, 10, 11, 12, 13 |
| Generalisability | 21 | Discuss the generalisability (external validity) of the study results | 13 |
| **Other information** | | | |
| Funding | 22 | Give the source of funding and the role of the funders for the present study and, if applicable, for the original study on which the present article is based | 13 |

*Give information separately for exposed and unexposed groups.

**Note:** An Explanation and Elaboration article discusses each checklist item and gives methodological background and published examples of transparent reporting. The STROBE checklist is best used in conjunction with this article (freely available on the Web sites of PLoS Medicine at http://www.plosmedicine.org/, Annals of Internal Medicine at http://www.annals.org/, and Epidemiology at http://www.epidem.com/). Information on the STROBE Initiative is available at www.strobe-statement.org.
